# Digital Medical Information Services Delivered by Pharmaceutical Companies via WeChat: Qualitative Analytical Study

**DOI:** 10.2196/43812

**Published:** 2023-11-17

**Authors:** Ying Ge, Dongning Yao, Carolina Oi Lam Ung, Yan Xue, Meng Li, Jiabao Lin, Hao Hu, Yunfeng Lai

**Affiliations:** 1 State Key Laboratory of Quality Research in Chinese Medicine, Institute of Chinese Medical Sciences University of Macau Macao China; 2 School of Pharmacy Nanjing Medical University Nanjing China; 3 Department of Public Health and Medical Administration, Faculty of Health Sciences University of Macau Macao China; 4 Key Laboratory of Environmental Medicine Engineering of Ministry of Education, Department of Medical Insurance, School of Public Health Southeast University Nanjing China; 5 School of Public Health and Management Guangzhou University of Chinese Medicine Guangzhou China

**Keywords:** digital medical information service, pharmaceutical company, WeChat, social media, digital health

## Abstract

**Background:**

Social media has become one of the primary information sources for medical professionals and patients. Pharmaceutical companies are committed to using various social media platforms to provide stakeholders with digital medical information services (DMISs), which remain experimental and immature. In China, WeChat tops the list of popular social media platforms. To date, little is known about the service model of DMISs delivered by pharmaceutical companies via WeChat.

**Objective:**

This study aims to explore the emerging service model of DMISs delivered by pharmaceutical companies via WeChat in China.

**Methods:**

This study applied a qualitative research design combining case study and documentary analysis to explore the DMISs of 6 leading pharmaceutical companies in China. Materials were collected from their official WeChat platforms. Thematic analysis was conducted on the data.

**Results:**

The DMISs of 6 pharmaceutical companies were investigated. Themes emerged regarding 2 essential information services delivered by pharmaceutical companies via WeChat: business operation services and DMISs (ie, public information services, professional services, science and education services, and e-commerce services). Business operation services mainly function to assist or facilitate the company’s operations and development trends for general visitors. Public-oriented information services are realized through health science popularization, academic frontiers, product information, and road maps to hospitals and pharmacies. Internet hospital and pharmacy services are the main patient-oriented professional services. Medical staff–oriented science and education services commonly include continuing education, clinical assistance, academic research, and journal searching. Public-oriented e-commerce services include health products and health insurance.

**Conclusions:**

Pharmaceutical companies in China use WeChat to provide stakeholders with diversified DMISs, which remain in the exploratory stage. The service model of DMISs requires more distinct innovations to provide personalized digital health and patient-centric services. Moreover, specific regulations on the DMISs of pharmaceutical companies need to be established to guard public health interests.

## Introduction

### Background

Due to advancements in digital technology, social media has become one of the main digital medical information sources for patients and medical professionals [1,2]. It allows individuals and communities to gather and communicate with others [3]. Social media has changed how the general public accesses medical information and informs their health-seeking behavior [4]. Patients use social media to keep in touch with physicians, share their medical experiences, and exchange ideas and feelings on a specific medical topic [5]. In contrast, medical professionals use social media to develop professional networks, increase patients’ awareness of diseases and medicines, advocate for patients to receive appropriate and timely treatment, and provide general medical information to the community [6]. The process in which stakeholders use digital social media to obtain or provide medical information services is collectively referred to as digital medical information services (DMISs) in this study.

The mainstream social media platforms in China include Weibo, Xiaohongshu, TikTok, and WeChat [7]. It was found that DMISs are provided widely on these social media platforms in China. Regarding Weibo, 2 modes are used by users to process health information: the heuristic mode to repost, which is sensitive to public messages, negative appeals, and nonprofessional authority, and the systematic mode to endorse and reply, which is sensitive to private messages, positive appeals, and both professional and nonprofessional authorities [8]. Users prefer low-fear appeal messages for health information on Weibo and are less likely to accept messages containing efficacy information [9]. Weibo can contribute to the knowledge gaps in short-term individual changes in psychological conditions, which can assist policy-makers in developing actionable policies and help clinical practitioners (eg, social workers, psychiatrists, and psychologists) provide timely services to affected populations [10]. Weibo can help patients seek health information during a pandemic, which is especially beneficial for vulnerable groups [11]. It can also monitor user suicide risk and provide crisis interventions [12]. Although the health information interaction of Weibo runs smoothly with a profound effect on service users and providers, the degree of interaction is insufficient, and the network remains loose [13]. Xiaohongshu, one of China’s fastest-growing social media apps, encourages its users to post different content about their lives [14]. An increasing number of users interact with each other on this platform according to their interests in various domains, such as specific medicines, health products, and food [15,16]. Regarding TikTok, the health dissemination of the short video platform is becoming an emerging DMIS. However, some problems remain concerning the fact that the main bodies of health dissemination are generally nonprofessional, the authenticated names of the main bodies of dissemination are confusing and misleading for the audience, much health dissemination content is unscientific and endangers audience judgment, and short video production formats lack diversity [17].

WeChat, among others, is the most popular social media platform in China, and there are >1.27 billion monthly active user accounts [18,19]. WeChat is a free mobile app released by Tencent in 2011. In the past decade, WeChat has evolved to be a super app that provides multiple services to its users in their daily lives, including searching and adding friends directly via WeChat ID or mobile number, chatting freely, sending messages, sharing photos and videos, sharing moments, making free video and voice calls, and making mobile payments [20]. Because of its tremendous number of users and sufficient digital service capabilities, WeChat is gradually being used in professional areas such as medical education and patient follow-up [21,22]. For example, a web-based questionnaire survey found that, for 63.26% of respondents, WeChat was their first choice to obtain health education [23].

Obviously, DMISs are rapidly emerging on social media. As one of the key stakeholders in the health system, pharmaceutical companies use various social media platforms to deliver DMISs to different stakeholders. For example, pharmaceutical companies can improve their corporate reputation by actively managing their Facebook profiles [24]. Twitter has also been widely used in conferences by pharmaceutical companies to develop public relations with clinicians [25]. Although marketing and advertising activities on the social media platforms of pharmaceutical companies are regulated, they publicize nondrug treatment information, announcement news, job information, drug statements, and other information on Facebook, Twitter, and YouTube [26]. In China, many Chinese patent medicine companies use Weibo to provide information about their products, spread health information, and promote traditional Chinese medicine (TCM) culture [27]. Nowadays, digital medication health service platforms are increasingly being used by pharmaceutical companies to deliver direct medication health services in the country [28].

However, based on our previous research on the DMISs of pharmaceutical companies, there is currently a lack of in-depth research on this subject as well as research on pharmaceutical companies using WeChat, the most popular social media platform in China, to deliver DMISs. Specifically, existing research mainly focuses on the content or effectiveness of digital services of pharmaceutical companies but does not summarize their service models.

### Objectives

Therefore, it is of practical significance to investigate WeChat to analyze the DMISs of pharmaceutical companies comprehensively. This study aimed to explore the emerging service models of DMISs delivered by pharmaceutical companies via WeChat in China. It is expected that the findings of this study will contribute to generating references for further application and improvement of DMISs of pharmaceutical companies and provide evidence for the future development of specific regulations on DMISs via social media by pharmaceutical companies and other stakeholders.

## Methods

### Research Design

This study used a qualitative research design by combining case study and document analysis [29]. The READ (ready materials, extract data, analyze data, and distill findings) approach to document analysis was used in this study [30]. As DMISs by pharmaceutical companies are an emerging field and a standardized approach to investigate them is yet to be developed, qualitative research focusing on the practices of the leading pharmaceutical companies is necessary to generate exploratory evidence.

The stepwise approach of this study mainly included the following actions. First, we referred to the *Top 100 China National Pharmaceutical Industry in 2020* list to screen and select the sample pharmaceutical companies for further analysis in this study [31]. Second, we accessed the WeChat official accounts and linked mini programs on WeChat set up by the selected pharmaceutical companies to collect the information services they deliver. Third, we identified and classified all information services for further exploration. Each of these steps is explained in more detail in the following sections.

### Selection of Sample Pharmaceutical Companies

Multi-interpretation case selection was used in this study for sampling [32]. Multiple explanatory case studies have focused on explaining how and why phenomena occur. First, we selected sample pharmaceutical companies from the *Top 100 China National Pharmaceutical Industry in 2020* list published by the China National Pharmaceutical Industry Information Center on August 1, 2021. This list is formulated based on legal entities’ annual primary business income for the pharmaceutical industry. The China National Pharmaceutical Industry Information Center is subordinate to the Ministry of Industry and Information Technology of the People’s Republic of China, which is an official institution, and the information released by it is officially representative. Therefore, based on this list, the pharmaceutical companies we selected have sufficient ability and willingness to deliver DMISs. The list categorized the companies by capital type, namely, foreign-invested and local-invested companies, conventional medicine companies, and TCM companies.

For this study, to screen the pharmaceutical companies for inclusion, we first selected the top 10 local conventional medicine companies, the top 10 TCM companies, and the top 10 foreign pharmaceutical companies (Multimedia Appendix 1). Second, we identified the information services of these 30 pharmaceutical companies by checking their models of information service provision one by one through their official WeChat accounts and linked mini programs, and based on that, we categorized the business operation services and DMISs. Third, we reviewed the identified official WeChat accounts and mini programs of these 30 pharmaceutical companies using the following inclusion criteria: (1) the certification of the WeChat official account and mini program was completed, and they passed the annual audit conducted by Tencent and third-party audit institutions; (2) the official account and mini program services are accessible and free; (3) the official account and mini program services are updated at least once a month; and (4) there are no less than 2 DMISs.

### Data Collection and Analysis

Data were collected from the official WeChat platforms and linked mini programs of the selected pharmaceutical companies. A total of 90 official WeChat accounts and mini programs were selected. All the official WeChat accounts and mini programs of these 6 pharmaceutical companies are shown in Multimedia Appendix 2. Specifically, WeChat mini programs refer to the “subapplications” within the WeChat ecosystem that enable the provision of advanced features to users, such as e-commerce, online store tours, task management, coupons, and other services. The last search was conducted until September 10, 2021.

Data were collected from different domains listed on the WeChat official accounts of the included pharmaceutical companies, such as the service content, target customers, information-seeking processes, and sources of information quoted. Thematic analysis was used on the data to better understand the link between the targets and purposes of information provision via the DMISs [33]. First, 3 investigators analyzed the documentary materials extracted from each of the included DMIS WeChat platforms and developed the codes separately. Then, all the suggested codes were pooled, regrouped, and categorized into themes and subthemes by these 3 investigators. The coding system gave rise to a number of themes and subthemes that encompassed the content, target customers, processes, and resources. On the basis of these dimensions, a framework to categorize different types of DMISs was developed and agreed upon by 3 investigators and confirmed by 2 senior investigators. Second, 3 investigators analyzed each DMIS separately using the framework. They compared the similarities and differences among the included DMISs to reach a consensus on their categorization and the content of the information provided. Disagreements on the coding results were subject to final confirmation by 2 senior investigators.

### Ethics Approval

The study design was approved by the ethics committee of the University of Macau (approval SSHRE21-APP027-ICMS).

## Results

### Sample Selection and Characteristics

**Figure 1:**
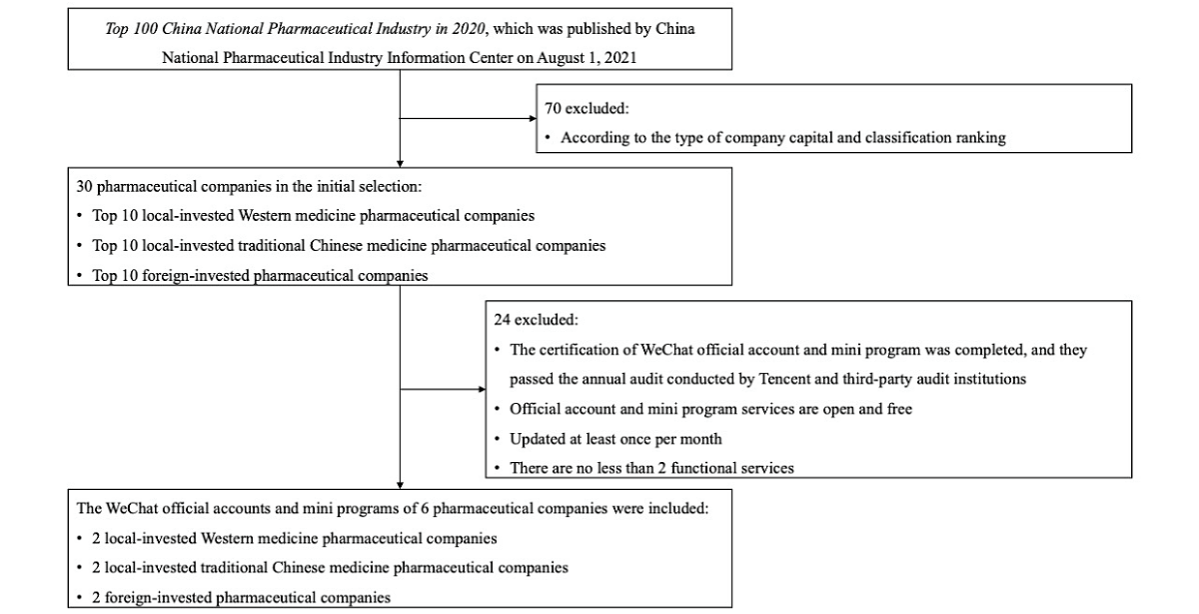
Flowchart of sample selection.

This study selected 6 pharmaceutical companies that met the inclusion criteria, including 2 (33%) local-invested Western medicine pharmaceutical companies, 2 (33%) local-invested TCM pharmaceutical companies, and 2 (33%) foreign-invested pharmaceutical companies. The flowchart of the sample selection process is shown in Figure 1.

The background information for the 6 sample pharmaceutical companies is shown in Table 1 (the details of the sample pharmaceutical companies are shown in Multimedia Appendix 3). As of September 10, 2021, the number of WeChat official accounts and mini programs of Jiangsu Hengrui Pharmaceuticals Co, Ltd (Hengrui); Shanghai Fosun Pharmaceutical (Group) Co, Ltd (Fosun); China Beijing Tongrentang (Group) Co, Ltd (TRT); Tasly Holding Group Co, Ltd (Tasly); AstraZeneca Pharmaceutical Co, Ltd (AZ); and Hangzhou Merck Pharmaceutical Co, Ltd (Merck) that were included in this study was 4, 9, 52, 19, 6, and 4, respectively.

**Table 1:** Background information on the sample pharmaceutical companies.

Company	Company type	Main business and products
Jiangsu Hengrui Pharmaceuticals Co, Ltd	Local conventional medicine company	Main business: drug R&D^a^, production, and sales Main products: antitumor drugs, surgical anesthetics, special infusions, contrast agents, cardiovascular drugs, among others
Shanghai Fosun Pharmaceutical (Group) Co, Ltd	Local conventional medicine company	Main business: pharmaceutical manufacturing, medical devices and medical diagnosis, and health care services Main products: cardiovascular, metabolic and digestive system, central nervous system, blood system, anti-infection and antitumor drugs or medications
China Beijing Tongrentang (Group) Co, Ltd	TCM^b^ company	Main business: focus on TCM production as its core pillar supplemented by 4 other pillars—health and wellness, senior medical care, commerce and retail, and international business—shaping a health industry chain that covers herb growing, TCM production, sales, medical services, health care, and R&D Main products: TCM, Chinese patent medicine, and prepared pieces of TCM
Tasly Holding Group Co, Ltd	TCM company	Main business: pharmaceuticals, preinspection, early warning, prevention, diagnosis, treatment, and rehabilitation Main products: cardiovascular, cerebrovascular, digestion and metabolism, tumor immunity, and neuroscience drugs or medications
AstraZeneca Pharmaceutical Co, Ltd	Foreign pharmaceutical company	Main business: the R&D, production, and sales of chemical preparations, chemical APIs^c^, antibiotics, biochemical drugs, and biological products Main products: cardiovascular, kidney and metabolism, tumor, respiratory, digestion, anesthesia, and neuroscience drugs or medications
Hangzhou Merck Pharmaceutical Co, Ltd	Foreign pharmaceutical company	Main business: drug R&D, production, and sales Main products: prescription drugs, vaccines, biopharmaceuticals, and animal health products

^a^R&D: research and development.

^b^TCM: traditional Chinese medicine.

^c^API: active pharmaceutical ingredient.

### Summary of Service Models for WeChat Information Services

**Figure 2:**
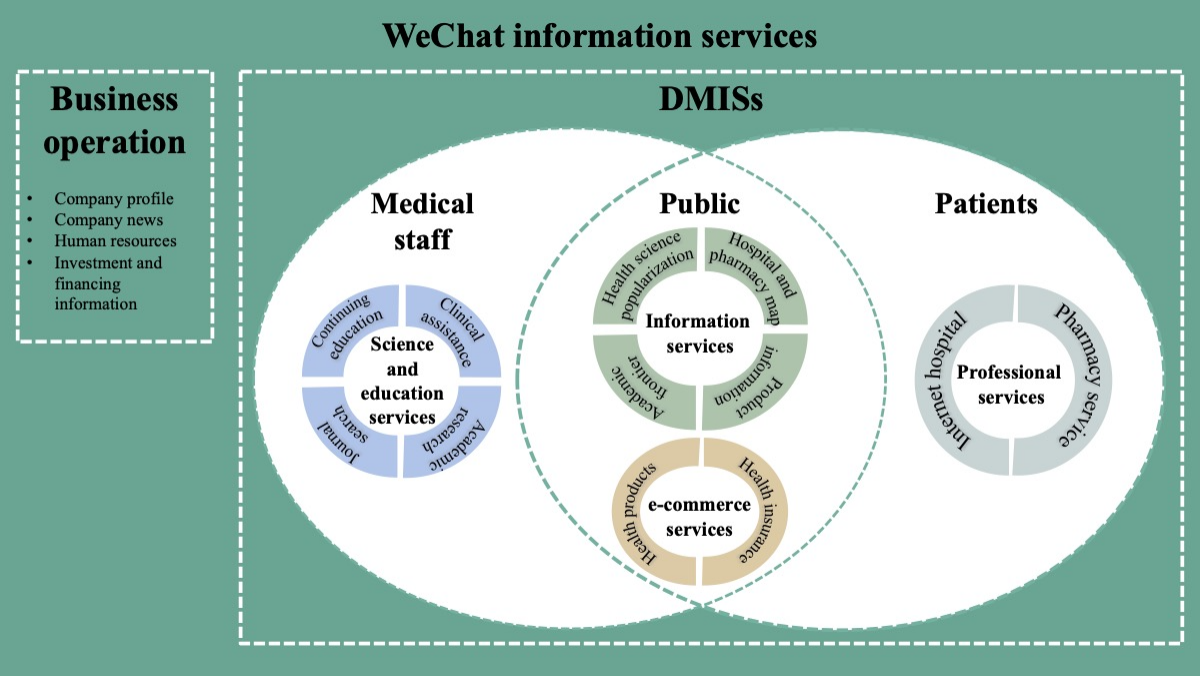
Full picture of WeChat information services. DMIS: digital medical information service.

The thematic analysis results gave rise to 3 main themes for the DMIS service model based on the target customers: medical staff, the public, and patients. Within each theme, a number of subthemes were identified based on the purposes of information provision: scientific updates and education, service- or product-based information, online shopping, and accessibility to professional health care services. In addition, all the WeChat information services can be divided into business operation services and DMISs. As shown in Figure 2, the target customer, value offering, and service provision of the service model were identified.

Business operation services delivered by pharmaceutical companies were public oriented. Business operation services mainly included functions to assist or facilitate the company’s operations and the development trends for general visitors. There were four subtypes of this service:

1. Company profiles to provide information on their business, history, and development. For example, TRT explained the company’s development history, corporate culture, and business scope through image, text, and video via the official WeChat account and mini program, which could help the public understand the company culture.
2. Company news to show trends and new developments. For example, Tasly shared brand activities, stories of excellent employees, and revenue status via the WeChat official account and mini program. Unlike the company profile, this service focused on reporting the company’s latest news.
3. Human resources information to provide personnel recruitment information to the public. Some companies built special WeChat official accounts and mini programs to deliver recruitment information. For example, “AstraZeneca Recruitment” provides subscribers with campus recruitment, social recruitment, and internship recruitment information.
4. Investment and financing information services to provide the public with information on investment and financing in the health industry. Only Tasly and AZ had these functions in place.

DMISs could be categorized into 4 types and 12 subtypes: public information services (health science popularization, academic frontier, product information, and road maps to hospitals and pharmacies), professional services (internet hospital and pharmacy service), science and education services (continuing education, clinical assistance, academic research, and journal search), and e-commerce services (health products and health insurance). The public information service and e-commerce services mainly served the public, whereas professional services were provided to patients and science and education services were offered to medical workers. The detailed WeChat information services delivered by the pharmaceutical companies are shown in Table 2. A specific analysis of the 4 types of DMISs delivered by the pharmaceutical companies via WeChat is provided in the following sections.

**Table 2:** Summary of WeChat information services delivered by pharmaceutical companies.

Service and subcategories				Hengrui^a^		Fosun^b^		TRT^c^		Tasly^d^		AZ^e^		Merck^f^
**Business operation services**														
	Company profile			✓		✓		✓		✓		✓		✓
	Company news			✓		✓		✓		✓		✓		✓
	Human resources			✓		✓				✓		✓		✓
	Investment and financing information									✓		✓		
**DMISs^g^**														
	**Science and education services**													
		Continuing education			✓		✓		✓		✓		✓	
		Clinical assistance									✓		✓	
		Academic research	✓								✓			
		Journal search									✓		✓	
	**Professional services**													
		Internet hospital			✓		✓		✓					
		Pharmacy service			✓		✓				✓			
	**Public information services**													
		Health science popularization	✓		✓		✓		✓		✓		✓	
		Academic frontier	✓								✓		✓	
		Product information	✓		✓		✓		✓		✓		✓	
		Hospital and pharmacy map					✓		✓		✓		✓	
	**e-Commerce services**													
		Health products			✓		✓		✓					
		Health insurance			✓									

^a^Hengrui: Hengrui Pharmaceuticals Co, Ltd.

^b^Fosun: Shanghai Fosun Pharmaceutical (Group) Co, Ltd.

^c^TRT: China Beijing Tongrentang (Group) Co, Ltd.

^d^Tasly: Tasly Holding Group Co, Ltd.

^e^AZ: AstraZeneca Pharmaceutical Co, Ltd.

^f^Merck: Hangzhou Merck Pharmaceutical Co, Ltd.

^g^DMIS: digital medical information service.

### First Type of DMIS: Science and Education Services for Medical Staff

#### Overview

Science and education services for medical staff included 4 subtypes: continuing education, clinical assistance, academic research, and journal search. The summary of science and education services is shown in Multimedia Appendix 4.

#### Continuing Education

Continuing education mainly provided services for medical staff to participate in regular professional examinations in terms of examination materials, examination outlines, examination registration, among other things. Fosun, TRT, AZ, and Merck provided physicians with professional web-based training courses such as academic conferences, clinical cases, and specialized medicine. Merck divided continuing education into tumor, diabetes, and vaccine areas; hospital specialist; and pharmacist college so that medical professionals could choose their training materials and learning mode according to their personal needs. Moreover, the clinical materials of the People’s Medical Publishing House, such as monographs, cases, drugs, and clinical guidelines, were also available on these platforms.

#### Clinical Assistance

Clinical assistance was designed to provide web-based medical index calculations (eg, general medical formulas and specialty indicators) and clinical guideline inquiries for clinicians. Only the 2 foreign-invested pharmaceutical companies, AZ and Merck, provided clinical assistance services. A medical index calculation service was used for the medical conversion of clinical indicators to quickly interpret their clinical significance. Both companies cooperated with Yimaitong to offer web-based medical index calculation services. Yimaitong is a platform covering medical information, case data, a medical knowledge base, and clinical guidelines. AZ cooperated with AskBob Doctor to provide interpretation of inspection or laboratory reports and address queries about clinical medication guidelines and drug interaction databases. AskBob Doctor is a consultation and treatment assistance tool supporting research, diagnosis, and treatment decisions that is a key driver of Ping An’s health care ecosystem. Merck used artificial intelligence technology to provide clinical assistance services. The clinical diagnosis and treatment knowledge it provided was divided into a professional version and a public version.

#### Academic Research

Academic research was designed to provide services for clinical research, such as web-based patient recruitment and web-based questionnaire surveys. Hengrui used the official WeChat account to recruit patients for its clinical trials. In collaboration with AskBob Doctor, AZ built an open medical communication platform. Those who joined could independently initiate questionnaires on the platform.

#### Journal Search

Journal search provided both Chinese and English academic literature search services. Cooperating with Elsevier, PubMed, Wangfan Data, and AskBob Doctor, AZ and Merck provided WeChat search services of mostly academic literature to medical staff. In addition, AZ cooperated with Nucleus Global, a specialized medical communication company, to provide publication planning and scientific editing support services to help users screen, select publishers, and contribute.

### Second Type of DMIS: Professional Services for Patients

#### Overview

Professional services for patients included 2 subtypes: internet hospital and pharmacy services. The summary of professional services is shown in Multimedia Appendix 5.

#### Internet Hospital

The internet hospital was designed to provide closed-loop medical services (ie, web-based and offline medical services, including web-based registration, web-based medical consultation, web-based drug supply and support, and web-based payment services) to patients using the medical resources of brick-and-mortar hospitals and internet technology. Internet hospitals were divided into patient end and physician end according to different users. For example, the Fosun and Tasly internet hospitals provided general web-based medical services, whereas the TRT internet hospital provided web-based TCM services.

#### Pharmacy Service

The pharmacy service was for pharmacists to guide patients in rational drug use, including drug-related indications, use, dosage, and adverse reactions. TRT provided web-based consulting services on TCM with pharmacists. AZ provided web-based medication consulting services with professionals.

### Third Type of DMIS: Public Information Services for the Public

#### Overview

Public information services for the public included 4 subtypes: health science popularization, academic frontiers, product information, and road map to hospitals and pharmacies. The summary of public information services is shown in Multimedia Appendix 6.

#### Health Science Popularization

Health science popularization was a common function of the DMISs to provide health science knowledge to the public via articles and videos, including health care knowledge, disease knowledge, and medication knowledge, which might be based on modern and TCM theories. For example, the public could obtain epidemic-related knowledge of COVID-19 and mRNA vaccines on Fosun’s official WeChat account and health knowledge of TCM, especially the compatibility of medicine and food, on TRT’s official WeChat account. On Merck’s official WeChat account, the professional and public vaccine and disease prevention knowledge versions were designed for health professionals and the public, respectively.

#### Academic Frontiers

The function of academic frontiers is to provide the public with the latest scientific research results and academic activity information via articles and videos. For example, Hengrui released the latest scientific research results and academic conferences on lung and breast tumors, urinary blood tumors, and other diseases in its official WeChat account. AZ announced the clinical research and development progress of the company’s drugs via the WeChat platform.

#### Product Information

Product information was designed to provide the public with drug and medical device instructions and regulatory information. AZ allowed its users to scan the barcode on the drug packaging box through WeChat to inquire about the drug instructions, disease knowledge, and some common medication problems. TRT provided a code-scanning query function to verify the authenticity information on specific products such as the angong niuhuang pill.

#### Road Map to Hospitals and Pharmacies

The road map to hospitals and pharmacies was designed to provide the public with hospital or pharmacy address query and navigation services. This function contained 2 service paths: one was to inquire about hospitals or pharmacies through products and the other was to inquire about products through hospitals or pharmacies. Its purpose was to improve the convenience of users’ access to drugs or medical services. For example, TRT and Tasly cooperated with Tencent maps to provide users with map services to find pharmacies or hospitals in their nearby area. The approach taken by AZ and Merck was more product oriented, which helped patients locate hospitals or pharmacies selling their products through this function.

### Fourth Type of DMIS: e-Commerce Services for the Public

#### Overview

e-Commerce services for the public included 2 subtypes: health products and health insurance. A summary of e-commerce services is shown in Multimedia Appendix 7.

#### Health Products

Health product e-commerce services sell daily necessities, food, health products, drugs, medical devices, and medical service packages on the web. TRT established the Tongrentang Internet Hospital Mall, which provides online purchasing services for food, health products, and over-the-counter drugs. Cross-border online purchase services of international food and health products are available to members on the TRT International Mall. Tasly’s health product service sold both over-the-counter and prescription drugs on the web.

#### Health Insurance

Health insurance was an innovative service in e-commerce services. For example, Fosun provided an all-inclusive insurance service covering medical insurance, sickness insurance, disability income loss insurance, nursing care insurance, accident insurance, and other insurance services. Customers could complete all processes, such as health insurance consultation, purchase, renewal, and claim settlement, in the WeChat official account and mini program.

## Discussion

### Principal Findings

This study reports a detailed description of DMISs delivered by pharmaceutical companies via WeChat in China. We investigated the business operation services and DMISs of 6 sample pharmaceutical companies. Although the pharmaceutical companies used many technology-enabled systems and services, the scale of provision and the types and capabilities of DMISs differed considerably across the companies. The understanding of these DMIS aspects in the existing literature needs to be improved. Our findings could contribute to understanding the service model of DMISs, which consisted of 4 types and 12 subtypes involving the target customer, value offering, and service provision. On the basis of our results, some points are worthy of further discussion in the following sections.

### Pharmaceutical Companies Delivering Multiple DMISs

The literature indicates that DMISs could improve physician-patient communication, patient health management, and pharmacy services [5,34-36]. However, we found that pharmaceutical companies delivered multiple DMISs via WeChat, which differed greatly. DMISs provided not only professional services for patients (internet hospital and pharmaceutical care) but also auxiliary services for physicians (clinical assistance and academic research) and e-commerce services for the public (health products and health insurance). Compared with foreign digital health services, the DMISs of pharmaceutical companies in China were broader in terms of both content and audience. Furthermore, there were noticeable differences among the pharmaceutical companies from a service content perspective. Professional services and e-commerce services were the main features of local-invested pharmaceutical companies, whereas foreign-invested pharmaceutical companies preferred to provide science and education services through DMISs. As they had different corporate strategies and corporate cultures, the service design of the companies in different countries also varied [37,38].

### Purposes of Pharmaceutical Companies Delivering DMISs

The gap between health care demand and medical resource supply in China and worldwide is widening [39]. Moreover, people are no longer prepared to be passive care recipients but expect health care services to be available when and where needed [40]. Therefore, digital transformation is crucial in helping bridge the gap [41-43]. In China, many policy interventions have been introduced in the “Internet plus Medical Care and Health” sector to support the development of digital health over the past 15 years [44]. Pharmaceutical companies are among the core stakeholders of the health system and are responsible for the research and development, production, and sale of drugs. However, these responsibilities no longer align with market development trends, and the companies can assume more social and corporate responsibilities [45]. After collating and summarizing the DMISs delivered by pharmaceutical companies in China, it became apparent that the digital transformation of China’s medical services has become a significant trend [46]. It helps meet the patients’ health care demands and improves the efficiency of health care service supply [47].

After systematically investigating the pharmaceutical companies’ DMISs, we found that the companies actively developed the information service capability of their WeChat official accounts and mini programs. The pharmaceutical companies were aware of the benefits of digital technology in improving user experience and reducing costs, which could help them establish a positive image and expand their social influence [48]. In fact, many of them had tried to develop services on the WeChat platform in the past. However, owing to the influence of traditional marketing models and the lack of successful business models that could be used for reference, the development often failed to achieve the expected results, and the pharmaceutical companies were still exploring the business model of DMISs [49].

### Potential Challenges of DMISs for Public Health

There are some challenges of DMISs for public health that need to be further discussed. First, companies may provide biased reports of their products. The selective reporting of information by pharmaceutical companies, such as in information services, may affect the initiative of users in information processing, especially medical professionals. Second, the boundaries of the DMISs provided by pharmaceutical companies for different target groups need to be clarified, and the content and readers need systematic matching, which affects the quality of information dissemination [36]. Third, there is a lack of practical clinical medication cases in existing DMISs, which are in high demand by medical staff. Finally, the current WeChat official accounts and mini programs provide fewer information services for nursing staff. Nevertheless, DMISs are very important for chronic disease management, in which nurses play an essential role [50].

In addition, regulatory challenges must be addressed. Although DMISs are an emerging information service, paying attention to their regulations is still important. In the United States, pharmaceutical companies must follow specific regulations when providing digital medical information to the public [51]. These include ensuring that all information is truthful and nonmisleading, complying with Health Insurance Portability and Accountability Act privacy and security regulations, and obtaining necessary approvals before distributing material. In addition, promotional activities have limitations that require strict adherence to Federal Trade Commission and Food and Drug Administration guidelines for fair balance and scientific accuracy. Ultimately, any content provided digitally must follow Food and Drug Administration guidance and regulations to ensure public safety and the appropriate use of prescription medicine. In Europe, one of the most significant pieces of legislation under the European Medicines Agency purview is Regulation (EC) 726/2004 (“Community Regulation”), which is applicable to pharmaceutical companies [52]. In China, regulations include the Cybersecurity Law of the People’s Republic of China [53], the Data Security Law of the People’s Republic of China [54], and advertising regulation policies. There is currently a lack of specialized legislation for DMISs, but individual countries are exploring new frameworks to complement existing regulatory treaties [55-57].

### Suggestions for Policy and Practice

In general, this study found that pharmaceutical companies have delivered many DMISs in China, but the value of DMISs has not been fully realized in operation. Thus, we propose the following suggestions for further development of DMISs. First, government supervision of information security is necessary. There is a need to introduce specific health information measures at the national level as implementation guidance for pharmaceutical companies and WeChat. Second, pharmaceutical companies should strengthen cooperation between different stakeholders to help expand DMISs, improve DMIS quality, and focus on audience-oriented DMISs. The tight coordination of engineering, data science, health IT, and clinical research functions would be more in line with the development needs of emerging DMISs. Third, the use of DMISs delivered via WeChat is suggested to develop and adopt safe and effective digital biomarkers to improve patient outcomes. To ensure privacy and autonomy, data use agreements for digital biomarkers should contain clear statements on data use conditions, especially for near-continuous data such as movement, voice, and other sensitive biometric states.

### Limitations

To the best of our knowledge, this is the first study investigating the DMISs delivered by pharmaceutical companies via WeChat. Several research limitations could be addressed in future studies. First, this study only conducted a analysis of the literature on DMISs presented by pharmaceutical companies on the WeChat platform. However, some documents need to be more detailed, and there was even a situation in which the information could not be retrieved. Future studies could focus on the actual operating data of the platform, which will be helpful to enhance our understanding of the DMISs. Second, there needs to be a more comparative analysis between the real needs of users and the service status. Third, this study needs to collect and analyze the opinions of digital information providers and receivers, and the results may be biased. It is necessary to conduct interviews with stakeholders of DMISs to strengthen the understanding of the DMISs from the perspectives of developers, users, and regulators.

### Conclusions

This study qualitatively explored the content, purposes, and suggestions of DMISs delivered by pharmaceutical companies via WeChat in China. Pharmaceutical companies in China use WeChat to provide diversified DMISs to stakeholders, which remain in the exploratory stage. The service model of DMISs requires more distinct innovations to provide personalized digital health and patient-centric services. Moreover, specific regulations on the DMISs of pharmaceutical companies need to be established to guard public health interests.

## Appendix

Multimedia Appendix 1 Top 10 pharmaceutical companies in different categories (ranking by the China National Pharmaceutical Industry Top 100 in 2020).

Multimedia Appendix 2 Details of the digital medical information services of the pharmaceutical companies.

Multimedia Appendix 3 Details of the sample pharmaceutical companies (ranking by the China National Pharmaceutical Industry Top 100 in 2020).

Multimedia Appendix 4 Summary of science and education services.

Multimedia Appendix 5 Summary of professional services.

Multimedia Appendix 6 Summary of public information services.

Multimedia Appendix 7 Summary of web-based mall services.

## Abbreviations

AZ: AstraZeneca Pharmaceutical Co, Ltd
DMIS: digital medical information service
Fosun: Shanghai Fosun Pharmaceutical (Group) Co, Ltd
Hengrui: Hengrui Pharmaceuticals Co, Ltd
Merck: Hangzhou Merck Pharmaceutical Co, Ltd
READ: ready materials, extract data, analyze data, and distill findings
Tasly: Tasly Holding Group Co, Ltd
TCM: traditional Chinese medicine
TRT: China Beijing Tongrentang (Group) Co, Ltd

## Data Availability

The data sets generated for this study are available from the corresponding authors upon reasonable request.
