# Supplementary material for: Digital Medical Information Services Delivered by Pharmaceutical Companies via WeChat: Qualitative Analytical Study
Source: J Med Internet Res. 2023 Nov 17;25:e43812. doi: 10.2196/43812 (PMC10692881; doi:10.2196/43812)
Supplement: Multimedia Appendix 1 [file jmir_v25i1e43812_app1.docx]

Multimedia Appendix 1. Top 10 pharmaceutical companies in different categories (ranking by the *China National Pharmaceutical Industry Top 100 in 2020*)

| Type | Ranking | Company |
| --- | --- | --- |
| Local-invested Western medicine pharmaceutical company | 1 | China National Pharmaceutical Group Co., Ltd. |
|  | 2 | Yangze River Pharmaceutical Group Co., Ltd. |
|  | 3 | Jiangsu Hengrui Pharmaceutical Co., Ltd. |
|  | 4 | China Resources Pharmaceutical Group Limited |
|  | 5 | Shanghai Fosun Pharmaceutical (Group) Co., Ltd |
|  | 6 | Shanghai Pharmaceutical (Group) Co., Ltd |
|  | 7 | Qilu Pharmaceutical Group Co., Ltd |
|  | 8 | CSPC Holdings Group Co., Ltd. |
|  | 9 | Zhengda Tianqing Pharmaceutical Group Co., Ltd |
|  | 10 | Sichuan Kelun Pharmaceutical Co., Ltd |
| Local-invested Chinese medicine pharmaceutical company | 1 | Guangzhou Pharmaceutical Group Co., Ltd |
|  | 2 | Xiuzheng Pharmaceutical Group Co., Ltd |
|  | 3 | Jiangxi Jimincare Group Co., Ltd |
|  | 4 | China Broad Group Co., Ltd. |
|  | 5 | Shandong Buchang Pharmaceutical Co., Ltd |
|  | 6 | Shijiazhuang Yiling Pharmaceutical Co., Ltd |
|  | 7 | Tianjin Pharmaceutical Group Co., Ltd |
|  | 8 | China Beijing Tongrentang (Group) Co., Ltd |
|  | 9 | Tasly Holding Group Co., Ltd. |
|  | 10 | Tianjin Hongri Pharmaceutical Co., Ltd |
| Foreign-invested pharmaceutical company | 1 | Bayer Healthcare Co., Ltd. |
|  | 2 | AstraZeneca Pharmaceutical Co., Ltd. |
|  | 3 | Pfizer Pharmaceutical Co., Ltd. |
|  | 4 | Novo Nordisk (China) Pharmaceutical Co., Ltd. |
|  | 5 | Shanghai Roche Pharmaceutical Co., Ltd. |
|  | 6 | Hangzhou Merck Pharmaceutical Co., Ltd. |
|  | 7 | Sanofi (China) Investment Co., Ltd. |
|  | 8 | Xi'an Janssen Pharmaceutical Co., Ltd. |
|  | 9 | Beijing Novartis Pharmaceutical Co., Ltd. |
|  | 10 | Fresenius Kabi (China) Investment Co., Ltd. |
