# Supplementary material for: Digital Medical Information Services Delivered by Pharmaceutical Companies via WeChat: Qualitative Analytical Study
Source: J Med Internet Res. 2023 Nov 17;25:e43812. doi: 10.2196/43812 (PMC10692881; doi:10.2196/43812)
Supplement: Multimedia Appendix 2 [file jmir_v25i1e43812_app2.docx]

Multimedia Appendix 2. Details of the digital medical information services of the pharmaceutical companies.

| **Company** | **WeChat** | **Account** | **Services** |
| --- | --- | --- | --- |
| Jiangsu Hengrui Pharmaceuticals Co., Ltd (Hengrui) | Hengrui mecial | hryy-dyh | - Hengrui information - Product system - Micro interaction |
|  | Hengrui recruitment | Hryy_Recruitment | - Explore Hengrui - Excellent mentor - Infinite possibility |
|  | Hengrui On Call | gh_a8b452ff6036 | - About Hengrui - Academic zone - Activity area |
|  | Jiangsu Hengrui clinical R & D information platform | hryylinchuang | - Content column - Patient recruitment - Expert channel |
| Shanghai Fosun Pharmaceutical (Group) Co., Ltd (Fosun) | Fosun | fosun-group | - Fosun vaccine - See 0656 - Sun community |
|  | Fosun United Health Insurance | Fosun-uhi | - Health insurance |
|  | Fosun Pharmaceutical Group recruitment | fosunPharma_HR | - Join us |
|  | Fosun United health insurance exhibition assistant | gh_a6bcc4b30a2a | - Micro insurance |
|  | Fosun pharmaceutical | Fosunpharma | - Sun dynamic - Sun gather - Sun recruit |
|  | Fosun health doctor terminal | Youyibang-y | - Patient management - Academic research - Mine |
|  | Fosun Health Home Edition | Youyibang-h | - Health topics - Medical services - Mine |
|  | Fosun diagnosis | gh_d87599425a28 | - Focus on diagnosis - Product matrix - I love FD |
|  | Fosun dental medical system | foshiondental | - Sun information - Sun store - Sun service |
| China Beijing Tongrentang (Group) Co., Ltd (TRT) | Tongrentang Internet hospital | TRTGJTCM | - Click follow-up - Mine - Report query |
|  | Beijing Tongrentang chain hospital | bjtrtlsyd | - Information query - Family pharmacy |
|  | Beijing Tongrentang terminal query | gh_82fd1e80f80d  (AppID:wxb1d57eb1ce50c793) | - Retail terminal - Medical terminal |
|  | Tongrentang health member | trtjkvip | - Integral benefits - Brand activities - Healthy kitchen |
|  | Beijing Tongrentang Hospital of traditional Chinese Medicine | tongrentangzyyy | - Doctor introduction - Hospital information - Patient service |
|  | Tongrentang optimization | TRTCARE | - Enter the mall - Registered member - Personal center |
|  | Tongrentang E member | trtyyhyzxgzh | - Medical services - Healthy knowledge - Member center |
|  | Tongrentang International - natural Amoy | tianrantaov | - Enter the mall - Global selection - Personal center |
|  | Tongrentang health | TRTJK-365 | - OUR - KNOW - HEALTH |
|  | Beijing Tongrentang Technology | tongrentang1669 | - Pharmacy culture - Your pharmacy - Pharmacy service |
|  | Tongrentang medical shop | tongrentanglaoyaopu | - Registration reservation - Healthy class - Service for you |
|  | Beijing Tongrentang Foshan Co., Ltd | tongrentangfs | - Corporate style - Convenient service - Member center |
|  | Beijing Tongrentang Shanxi chain store | tongrentangsxls | - Member center - Drug distribution - Pharmacist consultation |
|  | Beijing Tongrentang Angong Niuhuang Pill | trtanniu | - About us - Nearby drugstore - Authenticity query |
|  | Tongrentang healthy and good life | gh_9554047836c1 | - Healthy life - Healthy mall - Member service |
|  | Beijing Tongrentang Zhengzhou drugstore | bjtrtzzyd | - Seek medical advice - Publicity and Education - Member center |
|  | Beijing Tongrentang Zhongshan drugstore | bjtrtzsyd | - Tongrentang - Registration reservation - My appointment |
|  | Beijing Tongrentang Yanbian chain drugstore | trtybyd | - Member center - Micro mall - Pharmacy related |
|  | Beijing Tongrentang Shandong Pharmaceutical Chain Co., Ltd | trtsdls | - Centenarian shop - Famous doctors and drugs - Video customer service |
|  | Beijing Tongrentang health fan'er | trtjk_fan | - Live broadcasting room - Member mall - Member center |
|  | Tongrentang pharmaceutical | bjtrtzy | - Brand culture |
|  | Official WeChat of Tongrentang, Beijing, China | ZGBJTRTGW | - Brand - Product - Service |
|  | Beijing Tongrentang Shanxi online pharmacy | trtwsyd | - Micro mall - Online consultant - Personal center |
|  | Beijing Tongrentang Weihai Co., Ltd | bjtrtwh | - Tongrentang - Product service - Famous doctor |
|  | Beijing Tongrentang health new service micro-Journal | gh_75fdfc7d8901 | - About us - Service entrance |
|  | Beijing Tongrentang Linfen chain drugstore | bjtrtlflsyd | - Self-service - About us |
|  | Beijing Tongrentang Shijiazhuang traditional Chinese medicine hospital | trtsjzzyy | - Hospital introduction - Member welfare - Medical service |
|  | Chinese Beijing Tongrentang | gh_8b9fb849efa9 | - Service staff - Terminal query - Previous highlights |
|  | Beijing Tongrentang Foshan traditional Chinese Medicine Museum | trtfszyg | - / |
|  | Tongrentang healthy and good life | gh_9554047836c1 | - Healthy life - Healthy mall - Member service |
|  | Tongrentang international | trt-hi | - About us - Enterprise dynamics |
|  | Beijing Tongrentang Henan chain drugstore | bjtrthnls | - Follow up visit - Registration reservation - Personal center |
|  | Beijing Tongrentang Qinhuangdao drugstore | bjtrtqhdyd | - Micro member |
|  | Beijing Tongrentang meierhai Cosmetics | TRT-MIRAHI | - Service consultation - Mall Homepage |
|  | Beijing Tongrentang Yangquan pharmacy Co., Ltd | bjtrtyqyd | - Tongrentang - Traditional Chinese Medicine Museum - Self-service |
|  | Beijing Tongrentang medicinal material ginseng antler Investment Group | bjtrtycsrtzjt | - Product information - More wonderful |
|  | Beijing Tongrentang Sichuan health Co., Ltd | bjtrtjkj | - Star products - Wonderful Tongren - Health care colleagues |
|  | Beijing Tongrentang Xining drugstore | bjtrtxnyd | - Join us - National Medical Museum - More wonderful |
|  | Beijing Tongrentang health Fujian chain | TRTJK-FZLS | - Tongrentang - Member center - Wonderful content |
|  | Tongrentang Suzhou Street Medicine | trtdzmyd | - Micro reservation |
|  | Beijing Tongrentang Ningbo store | trt-nb | - About us - Registration reservation - Member acticity |
|  | Beijing Tongrentang Guangzhou chain store | bjtrtgz | - Online store - Drugstore |
|  | Beijing Tongrentang Hong Kong Traditional Chinese Medicine | bjtrtcmcl | - Tongren culture - Health Express - Online mall |
|  | Beijing Tongrentang Tianjin chain drugstore | bjtrttjyd | - Tongren boutique - Tongren Medical Museum |
|  | Beijing Tongrentang Co., Ltd | gh_acda999e1b7f | - Xiaotang information - Xiaotang Materia medica - Xiaoyang selection |
|  | Beijing Tongrentang Heilongjiang chain | bjtrthljlsydyxzrgs | - corporate culture - Member center |
|  | Beijing Tongrentang Jilin ginseng antler herbal workshop | bjtrt_1669 | - Capsule (Cordyceps) |
|  | Beijing Tongrentang Taiyuan traditional Chinese medicine hospital | trttyzyy | - Hospital address |
|  | Beijing Tongrentang Langfang drugstore Co., Ltd | bjtrtlfyd568 | - Registration reservation - Store navigation |
|  | Beijing Tongrentang Chaoyang drugstore chain Co., Ltd | bjtrtcyls | - Centenarian shop - Health care |
| Tianshili Holding Group Co., Ltd (TASLY) | Tasly big health | taslydajiankang | - Tasly - Enterprise dynamics - Healthy mall |
|  | Tasly big pharmacy | taslydrugstore | - Find and buy medicine - Nearby stores |
|  | Tasly pharmacy | taslypharma | - Innovation dynamics - Treatment area - About us |
|  | Tasly holding group | taslyfuwu | - News information - Innovation-driven development - Brand zone |
|  | Tasly capital | gh_eb88cf023654 | - Industry dynamics - Ecological enterprise - About us |
|  | Flagship store of Tasly pharmacy | taslydyf | - Healthy mall - Exclusive QR code - Mine |
|  | Tasly pharmaceutical business | tasly-yysy | - Mobile OA |
|  | Tasly yikang home | taslymbjkdj | - Digital chronic disease - Healthy mall - Personal center |
|  | Tasly research institute | tasly_academy | - Research institute |
|  | Tasly health training college | TASLY-peixunxueyuan | - Training news - Training source - Online study |
|  | Tasly health city | gh_0fb748e6648f | - Visiting service - Tourist attractions - Personal center |
|  | Tasly pharmaceutical business health | taslyhealth | - Training clock |
|  | Tasly public welfare foundation | taslyfoundation | - Public welfare project - About us |
|  | Tianjin Tasly pharmacy | gh_46b475afaad3 | - 580 consultation - Member service |
|  | Tianjin Beichen Tasly hospital | gh_0c1f3561f83a | - Hospital information - Medical examination center - Internet hospital |
|  | Yunnan Tasly Panax notoginseng pharmaceutical Co., Ltd | TASLY99 | - About us - Order goods - More service |
|  | Tasly health class | taslyjkkt | - Clock - Articles collection - Exclusive sales query |
|  | Tasly health recriutment | gh_58bdf06b2e77 | - Recruitment - Campus recruitment - Personal center |
|  | Benxi Taslty pharmacy service account | tasly_bx | - Member service - Deliver medicine home - Nearby drugstore |
| AstraZeneca Pharmaceutical Co., Ltd (AZ) | AstraZeneca China | AstraZenecaChina | - AZ headlines - Health talking - About us |
|  | AstraZeneca recruitment | gh_1fa6f9115165 | - Explore AZ - AZ recruitment - About AZ |
|  | AstraZeneca E medicine chest | gh_1ab67bb733ed | - Disease knowledge - Exclusive service - About us |
|  | AstraZeneca CICC medical industry fund | gh_fe4796f0d7f5 | - Invested enterprise |
|  | Yishitong | AZ_MedInfo | - Common tools - Medical service - Personal center |
| Hangzhou Merck Pharmaceutical Co., Ltd (Merck) | MSD China | msd_china | - Read story - Rising knowledge - Micro official website |
|  | MSD recruitment | msddazhao | - Understanding MSD - Join us |
|  | Uemeds | Uemeds | - Look - Search - Personal center |
