# Supplementary material for: Digital Medical Information Services Delivered by Pharmaceutical Companies via WeChat: Qualitative Analytical Study
Source: J Med Internet Res. 2023 Nov 17;25:e43812. doi: 10.2196/43812 (PMC10692881; doi:10.2196/43812)
Supplement: Multimedia Appendix 3 [file jmir_v25i1e43812_app3.docx]

Multimedia Appendix 3. Details of the sample pharmaceutical companies (ranking by the *China National Pharmaceutical Industry Top 100 in 2020*).

|  | Company | Company type | Company profile | Main business and products |
| --- | --- | --- | --- | --- |
| 1 | Jiangsu Hengrui Pharmaceuticals Co., Ltd (Hengrui) | Local-invested western medicine pharmaceutical company | Jiangsu Hengrui Pharmaceutical Co., Ltd. was founded in 1970 and listed on the Shanghai Stock Exchange in 2000. Its main business involves drug R & D, production and sales. Its main products cover many fields, such as antitumor drugs, surgical anesthetics, special infusion, contrast agents, cardiovascular drugs and so on. | - Main business: drug R & D, production and sales. - Main products: antitumor drugs, surgical anesthetics, special infusion, contrast agents, cardiovascular drugs and so on. |
| 2 | Shanghai Fosun Pharmaceutical (Group) Co., Ltd (Fosun) | Local-invested western medicine pharmaceutical company | Shanghai Fosun Pharmaceutical (Group) Co., Ltd. founded in 1994, is an innovation-driven international healthcare group in China. It operates businesses in the pharmaceutical and health industry, including pharmaceutical manufacturing, medical devices and medical diagnosis, and healthcare services. Through its associated company Sinopharm Co., Ltd., Fosun Pharma’s business extends to pharmaceutical distribution and retail. | - Main business: pharmaceutical manufacturing, medical devices and medical diagnosis, and healthcare services. - Main products: cardiovascular, metabolic and digestive system, central nervous system, blood system, anti-infection and anti-tumor |
| 3 | China Beijing Tongrentang (Group) Co., Ltd (TRT) | Local-invested Chinese medicine pharmaceutical company | Specializing in traditional Chinese medicine and therapy, Beijing TRT Group focuses on TCM production as its core pillar, supplemented by four other pillars of health and wellness, senior medical care, commerce and retail, and international business-shaping a health industry chain that covers herb growing, TCM production, sales, medical services, healthcare, and R&D. | - Main business: Focuses on TCM production as its core pillar, supplemented by four other pillars of health and wellness, senior medical care, commerce and retail, and international business-shaping a health industry chain that covers herb growing, TCM production, sales, medical services, healthcare, and R&D. - Main products: Traditional Chinese medicine, Chinese patent medicine and prepared pieces of traditional Chinese Medicine |
| 4 | Tasly Holding Group Co., Ltd (Tasly) | Local-invested Chinese medicine pharmaceutical company | Tasly Pharmaceutical Group Co., Ltd. is a modern Chinese medicine international pharmaceutical company that gathers major therapeutic areas such as cardiovascular, cerebrovascular, digestion and metabolism, tumor immunity, neuroscience, etc. The drug portfolio includes modern Chinese medicine, chemical medicine and innovative biological medicine. The business extends from pharmaceuticals to pre-inspection, early warning, prevention, diagnosis, treatment, and rehabilitation. | - Main business: The business extends from pharmaceuticals to pre-inspection, early warning, prevention, diagnosis, treatment, and rehabilitation. - Main products: Cardiovascular, cerebrovascular, digestion and metabolism, tumor immunity, neuroscience, etc. |
| 5 | AstraZeneca Pharmaceutical Co., Ltd (AZ) | Foreign-invested pharmaceutical company | AstraZeneca is a global biopharmaceutical company that focuses on cardiovascular, kidney and metabolism, tumor, respiratory, digestion, anesthesia, and neuroscience. | - Main business: It is mainly engaged in the R & D, production and sales of chemical preparations, chemical APIs, antibiotics, biochemical drugs and biological products. - Main products: Cardiovascular, kidney and metabolism, tumor, respiratory, digestion, anesthesia, and neuroscience. |
| 6 | Hangzhou Merck Pharmaceutical Co., Ltd (Merck) | Foreign-invested pharmaceutical company | Merck is an innovative biopharmaceutical company, focusing on prescription drugs, vaccines, biopharmaceuticals and animal health products. At present, it has an R&D center and factory in China, realizing the integration of R&D, manufacturing and commercial operations. | - Main business: it has an R&D center and factory in China, realizing the integration of R&D, manufacturing and commercial operations. - Main products: Prescription drugs, vaccines, biopharmaceuticals and animal health products. |
