# Supplementary material for: Digital Medical Information Services Delivered by Pharmaceutical Companies via WeChat: Qualitative Analytical Study
Source: J Med Internet Res. 2023 Nov 17;25:e43812. doi: 10.2196/43812 (PMC10692881; doi:10.2196/43812)
Supplement: Multimedia Appendix 4 [file jmir_v25i1e43812_app4.docx]

Multimedia Appendix 4. Summary of science and education services

|  | Hengrui^a^ | Fosun^b^ | TRT^c^ | Tasly^d^ | AZ^e^ | Merck^f^ |
| --- | --- | --- | --- | --- | --- | --- |
| Continuing education | —^g^ | 1. To provide physicians with professional web-bsed training courses, such as academic conferences, clinical cases, and specialized medicine through Youyibang internet hospital | 1. To provide physicians with professional disease education through TRT TCM^h^ experts | 1. To provide training on health industry–related knowledge to Tasly employees, including business training and strategy training | 1. To provide professional medical training to registered physicians through image-text and video, including patient education, symptoms and stages, and daily care 2. To provide medical professionals with examination materials and information on regular professional examinations | 1. To provide medical professionals with clinical materials from the People’s Medical Publishing House, such as monographs, cases, drugs, and clinical guidelines 2. To provide medical professionals with training and learning through the tumor, diabetes, and vaccine areas; hospital specialist; and pharmacist college |
| Clinical assistance | — | — | — | — | 1. Co-operation with Yimaitong to provide online medical index calculations, including medical general formulas and specialty indicators 2. Co-operation with AskBob Doctor to provide interpretation of inspection or laboratory reports, queries of clinical medication guidelines, and queries of drug interaction databases 3. To provide medical professionals with clinical medicine question-and-answer services, which are provided by professionals based on clinical experience, clinical guidelines, or medical literature 4. To provide reporting channels for adverse drug events, including telephone, email, and online reporting 5. To provide physicians, nurses, pharmacists, and internal employees with queries of drug encyclopedias, including Western medicine, Chinese patent medicine, and TCM prescriptions | 1. Co-operation with Yimaitong to provide online medical index calculations, including medical general formulas and specialty indicators 2. Co-operation with Yimaitong to provide drug information, medication review, drug guidelines, and adverse drug reaction information 3. To provide clinical research, clinical guidelines, clinical tools, and academic conferences on cancer, diabetes, and anti-infection based on AI^i^ 4. To provide a professional and public version of clinical diagnosis and treatment knowledge for medical professionals and the general public, respectively |
| Academic research | 1. To recruit patients for clinical research of Hengrui products | — | — | — | 1. Co-operation with AskBob Doctor to provide a questionnaire survey service for professionals | — |
| Journal search | — | — | — | — | 1. Co-operation with Wanfang Data to provide Chinese literature query services 2. Co-operation with AskBob Doctor to provide English literature query services | 1. Co-operation with Elsevier, PubMed, and Wangfan Data to provide literature query services |

^a^Hengrui: Hengrui Pharmaceuticals Co., Ltd.

^b^Fosun: Shanghai Fosun Pharmaceutical (Group) Co., Ltd.

^c^TRT: China Beijing Tongrentang (Group) Co., Ltd.

^d^Tasly: Tasly Holding Group Co., Ltd.

^e^AZ: AstraZeneca Pharmaceutical Co., Ltd.

^f^Merck: Hangzhou Merck Pharmaceutical Co., Ltd.

^g^ —: not applicable.

^h^TCM: traditional Chinese medicine.

^i^AI: artificial intelligence.
