# Supplementary material for: Digital Medical Information Services Delivered by Pharmaceutical Companies via WeChat: Qualitative Analytical Study
Source: J Med Internet Res. 2023 Nov 17;25:e43812. doi: 10.2196/43812 (PMC10692881; doi:10.2196/43812)
Supplement: Multimedia Appendix 5 [file jmir_v25i1e43812_app5.docx]

Multimedia Appendix 5. Summary of professional services.

|  | Hengrui^a^ | Fosun^b^ | TRT^c^ | Tasly^d^ | AZ^e^ | Merck^f^ |
| --- | --- | --- | --- | --- | --- | --- |
| Internet hospital | —^g^ | 1. To provide web-based medical consultation services, counter checks, and prescriptions to patients 2. To provide services such as triage, guidance, image-text medical consultations, telephone medical consultations, video consultations, patient management, clinical research, live video broadcasts, and web-based meetings to physicians | 1. To provide patients with web-based registration, web-based medical consultation, web-based prescription, web-based drug supply, and health consultation of TCM^h^ hospitals and TRT pharmacies 2. To provide a 9-valent HPV^i^ vaccine reservation service to TRT members 3. To provide Australian members with web-based registration services for professional services in Australia 4. To introduce the areas of expertise and outpatient information of TRT TCM experts | 1. To provide web-based counter check services for outpatient special diseases and general outpatients 2. To provide patients with web-based health consultation, web-based prescription, and web-based interpretation of physical examination reports 3. Co-operation with the Tianjin Diabetes Association to provide screening services for diabetic retinopathy 4. To provide members with web-based medical consultation services with pharmacy physicians | — | — |
| Pharmacy service | — | 1. To provide web-based medication guidance to patients through Youyibang internet hospital | 1. To provide web-based medication guidance to patients through TRT internet hospital 2. To provide web-based consulting services on TCM with pharmacists | — | 1. To provide web-based medication consulting services with professionals | — |

^a^Hengrui: Hengrui Pharmaceuticals Co., Ltd.

^b^Fosun: Shanghai Fosun Pharmaceutical (Group) Co., Ltd.

^c^TRT: China Beijing Tongrentang (Group) Co., Ltd.

^d^Tasly: Tasly Holding Group Co., Ltd.

^e^AZ: AstraZeneca Pharmaceutical Co., Ltd.

^f^Merck: Hangzhou Merck Pharmaceutical Co., Ltd.

^g^—: not applicable.

^h^TCM: traditional Chinese medicine.

^i^HPV: human papillomavirus.
