# Supplementary material for: Digital Medical Information Services Delivered by Pharmaceutical Companies via WeChat: Qualitative Analytical Study
Source: J Med Internet Res. 2023 Nov 17;25:e43812. doi: 10.2196/43812 (PMC10692881; doi:10.2196/43812)
Supplement: Multimedia Appendix 6 [file jmir_v25i1e43812_app6.docx]

Multimedia Appendix 6**.** Summary of public information services.

|  | Hengrui^a^ | Fosun^b^ | TRT^c^ | Tasly^d^ | AZ^e^ | Merck^f^ |
| --- | --- | --- | --- | --- | --- | --- |
| Health science popularization | 1. To provide health knowledge of tumor-related diseases through image-text or video | 1. To provide epidemic-related knowledge of COVID-19 and mRNA vaccines 2. To provide knowledge of chronic disease management, such as purine, diabetes, and nephropathy through image-text or video | 1. To provide health knowledge of traditional Chinese medicine, especially the homology of medicine and food, through image-text and video 2. The traditional Chinese medicine experts explain the knowledge of common disease prevention and treatment through image-text or video. | 1. To provide health knowledge related to cardiovascular and cerebrovascular diseases, tumor immunity, digestion and metabolism, neuroscience and other diseases, and relevant drug knowledge through image-text or video | 1. To provide knowledge of pathology, treatment, drugs, disease management, and disease burden in the fields of tumors, cardiovascular disease and metabolism, kidneys, respiration, and digestion through image-text, video, or web-based lecture | 1. To provide daily health science popularization in the form of animation 2. To provide professional and public versions of vaccine and disease prevention knowledge for health professionals and the general public, respectively |
| Academic frontier | 1. To provide academic research progress and related academic activities on lung tumors, head and neck tumors, hepatobiliary pancreatic tumors, gastrointestinal tumors, breast tumors, urinary blood tumors, among others | —^g^ | — | — | 1. To provide health professionals with the clinical research progress of the company’s drugs | — |
| Product information | 1. To provide basic information on innovative drugs, generic drugs, and internationally certified drugs | 1. To provide instructions for the company’s drugs and medical devices | 1. To provide the features of the company’s products 2. To provide anticounterfeiting queries of TRT’s drugs and cosmetics | 1. To provide basic information on products 2. To provide anticounterfeiting queries of Tasly’s drugs | 1. To provide basic information on the company’s product | 1. To provide basic information on the company’s product |
| Hospital and pharmacy map | — | — | 1. Co-operate with Tencent maps to provide address query and navigation services for all pharmacies and hospitals in TRT | 1. Co-operate with Tencent maps to provide address query and navigation services for all Tasly pharmacy stores | 1. Co-operate with Tencent maps to provide address query and navigation services for surrounding pharmacies or hospitals, oriented to finding medicines | 1. Co-operate with Tencent maps to provide address query and navigation services for medical institutions that can administer vaccines |

^a^Hengrui: Hengrui Pharmaceuticals Co., Ltd.

^b^Fosun: Shanghai Fosun Pharmaceutical (Group) Co., Ltd.

^c^TRT: China Beijing Tongrentang (Group) Co., Ltd.

^d^Tasly: Tasly Holding Group Co., Ltd.

^e^AZ: AstraZeneca Pharmaceutical Co., Ltd.

^f^Merck: Hangzhou Merck Pharmaceutical Co., Ltd.

^g^—: not applicable.
