# Supplementary material for: Digital Medical Information Services Delivered by Pharmaceutical Companies via WeChat: Qualitative Analytical Study
Source: J Med Internet Res. 2023 Nov 17;25:e43812. doi: 10.2196/43812 (PMC10692881; doi:10.2196/43812)
Supplement: Multimedia Appendix 7 [file jmir_v25i1e43812_app7.docx]

Multimedia Appendix 7. Summary of web-based mall services

| . | Hengrui^a^ | Fosun^b^ | TRT^c^ | Tasly^d^ | AZ^e^ | Merck^f^ |
| --- | --- | --- | --- | --- | --- | --- |
| Health products | —^g^ | 1. To provide web-based drug purchase services through Fosun Health Cloud Mall 2. To provide online purchase services of various dental packages | 1. To provide online purchase services, such as wine, salt, health products, and food, through the TRT internet hospital mall 2. To provide online purchase services of OTC^h^ drugs, food, and health products through the Family Pharmacy 3. To provide online purchase services such as beauty or washing products, food, wine, salt, health products, and OTC drugs through the Tongrentang Youxuan Mall 4. To provide members with cross-border online purchase services of international food and health products through the Tongrentang International Mall 5. To provide online TCM^i^ purchase, TCM decoction, and delivery of door-to-door services for members in the region | 1. To provide online purchase services of Deepure products through the Deepure Health Mall 2. To provide online purchase services such as nourishing TCM, health products, wine, tea, and cosmetics through the Kangxianghui Health Mall 3. To provide online purchase services such as department stores, beauty/washing products, toys, maternal and infant products, medical devices, OTC drugs, prescription drugs, health products, and food through the Tasly Pharmacy Mall 4. To provide door-to-door drug distribution services through the Tasly Pharmacy and Meituan Home | — | — |
| Health insurance | — | 1. To provide medical insurance, disease insurance, disability income loss insurance, nursing insurance, accident insurance, and other insurance services to the public | — | — | — | — |

^a^Hengrui: Hengrui Pharmaceuticals Co., Ltd.

^b^Fosun: Shanghai Fosun Pharmaceutical (Group) Co., Ltd.

^c^TRT: China Beijing Tongrentang (Group) Co., Ltd.

^d^Tasly: Tasly Holding Group Co., Ltd.

^e^AZ: AstraZeneca Pharmaceutical Co., Ltd.

^f^Merck: Hangzhou Merck Pharmaceutical Co., Ltd.

^g^—: not applicable.

^h^OTC: over the counter.

^i^TCM: traditional Chinese medicine.
